# Commentary: Breaking the Silence on ‘Failing to Fail’

**DOI:** 10.1177/17449871261452905

**Published:** 2026-08-29

**Authors:** Anna Pålsson

**Affiliations:** University Lecturer in Nursing, Merited Teacher and Module Leader, Department of Nursing, Kristianstad University, Kristianstad, Sweden

**Commentary to:** Nurses’ experiences of ‘failing to fail’: barriers and enablers in clinical nursing education – a qualitative study (Lannon et al., 2026).

The paper addresses a long-standing and important issue in nursing education, the reluctance to fail underperforming pre-registration nursing students in clinical placements. The phenomenon of ‘failing to fail’ is not new. It has been discussed for more than two decades and remains highly relevant because it concerns not only student progression but also patient safety, professional standards and public trust in the nursing profession (Hughes et al., 2016).

In this qualitative descriptive study, the authors explore registered nurses’ perceptions of why ‘failing to fail’ occurs and what may enable more appropriate and transparent assessment in practice. The four themes identified in the paper namely *Unspoken Truth, Time Limitations, Barriers to Failing and Enablers*, offer a convincing account of the social, emotional and organisational complexity surrounding assessment in clinical education. The study shows clearly that failing a student is rarely a simple matter of individual judgement. Instead, assessment is shaped by culture, confidence, relationships, time pressures and the quality of collaboration between clinical settings and universities. This aligns with previous literature showing that reluctance to fail is influenced by emotional strain, lack of confidence, ambiguity in assessment and fear of consequences for both student and assessor (Hughes et al., 2016; Jervis and Tilki, 2011).

One of the most valuable aspects of this paper is its naming of assessment failure as an ‘unspoken truth’. That phrase captures something essential. In many practice settings, concerns about underperformance are not absent, but difficult to articulate openly. Instead, they may be delayed, softened or left insufficiently documented. This creates a culture in which avoidance becomes easier than direct action. The paper therefore makes an important contribution by breaking that silence and giving language to experiences that many assessors will recognise but may not always feel able to discuss.

This strongly resonates with my own experience. I have worked with assessment conversations in clinical education for 19 years and felt an immediate recognition when reading this paper. In my own reflections, I noted how accurately it captures the reality of difficult conversations, guilt, frustration and conflicting perspectives in placement assessment. The complexity of assessment in clinical practice settings lies not only in the final grade but also in everything that happens before that point, namely observation, feedback, documentation, uncertainty, support and repeated attempts to help the student succeed. The assessor is often responsible not only for the final judgement but also for the process leading up to it. That can create both moral strain and professional loneliness, especially if collegial or institutional support is weak or arrives too late.

At the same time, the paper avoids oversimplifying the problem. It does not present assessors as lacking commitment or courage. Instead, it shows that barriers to failing are embedded in human relationships and organisational realities. Time limitations, in particular, are highly significant. Without time for observation, feedback, dialogue and reflection, assessment becomes more fragile. Under such conditions, it is understandable that difficult judgements become harder to make and harder to sustain. This point is especially persuasive in contemporary healthcare environments, where increasing workload and staffing pressures can weaken the conditions required for robust student supervision and assessment.

The paper’s discussion of enablers is equally important. Supportive workplace culture, collaborative university relationships, regular training and clear guidance are identified as key factors that help assessors act appropriately. These findings are consistent with earlier work suggesting that assessors are more able to manage underperformance when expectations are explicit, documentation is strong and institutional backing is reliable (Elliott, 2016; Luhanga et al., 2008). This shifts the focus away from blaming individuals and towards strengthening the systems in which assessment takes place.

This paper will be useful to several audiences. Clinically practicing nurses may recognise their own dilemmas in the findings. Academic staff may use the paper to support discussions about fairness, transparency and student progression. Leaders in education and practice may find it helpful when reviewing how assessors are prepared and supported. The paper is especially relevant in settings where staff feel isolated in difficult assessment decisions, where raising concerns is experienced as risky or where institutional processes are unclear.

Overall, this is a timely and valuable study. It addresses a persistent challenge in nursing education with clarity and sensitivity. Most importantly, it does not simply expose the problem, it points towards ways forward. More transparent communication, better collaboration, clearer guidance and stronger support structures are all necessary if difficult assessment decisions are to be handled fairly, confidently and in the interests of both students and patient safety. For nurse educators, supervisors and assessors, this paper offers both recognition and direction.
